# Tinnitus-Related Functional and Perceptual Impairments Following COVID-19 Vaccination: An Online Multi-Domain Survey Study

**DOI:** 10.3390/audiolres15060164

**Published:** 2025-11-26

**Authors:** Anusha Yellamsetty, Gianmaris Fortuna, Egbe-Etu Etu, Shaowen Bao

**Affiliations:** 1 Department of Audiology, San José State University, San José, CA 95192, USA; 2 Department of Marketing and Business Analytics, San Josè State University, San José, CA 95192, USA; 3 Department of Physiology and Department of Otolaryngology—Head and Neck Surgery, University of Arizona, Tucson, AZ 85724, USA

**Keywords:** tinnitus, COVID-19 vaccination, hyperacusis, speech-in-noise perception, auditory function, psychological distress

## Abstract

**Background:** Tinnitus has been increasingly reported during the COVID-19 pandemic, following both infection and vaccination. While these reports suggest that pandemic-related factors may influence the onset or worsening of tinnitus, the perceptual characteristics and functional consequences of such cases remain poorly understood. This study examined the nature, severity, and communication-related impact of self-reported tinnitus following COVID-19 vaccination. **Methods:** A total of 770 adults who self-reported new or worsened tinnitus after vaccination completed a structured online survey between August 2021 and May 2023. Standardized instruments included the Tinnitus Functional Index (TFI), the Speech, Spatial and Qualities of Hearing Scale (SSQ), and visual analog scales assessing loudness discomfort and hyperacusis-related symptoms. Analyses included descriptive statistics, chi-square tests, *t*-tests, and correlation matrices. **Results:** Respondents reported moderate to severe tinnitus-related distress, with the greatest impacts on emotional control, sleep, and relaxation. Many described communication difficulties in noisy or multi-talker environments, despite relatively preserved spatial hearing and sound quality. A substantial subset endorsed hyperacusis symptoms, including sound-induced annoyance, fear, and intolerance. Correlation analyses revealed strong associations between emotional, cognitive, and auditory domains, underscoring the multidimensional burden of tinnitus in this population. **Conclusions:** Self-reported tinnitus after COVID-19 vaccination was associated with distress, sleep disruption, and communication difficulties, mirroring patterns commonly observed in tinnitus more broadly. Although causality cannot be determined, these findings highlight the importance of comprehensive audiological and psychological assessment for individuals reporting auditory complaints after vaccination and support the inclusion of functional hearing outcomes in tinnitus evaluation protocols.

## 1. Introduction

Tinnitus, characterized by the perception of sound in the absence of an external source, affects an estimated 10–15% of the adult population [1]. These phantom auditory sensations—including ringing, buzzing, and hissing—can vary in pitch, loudness, and location, and are often associated with cochlear damage or sensorineural hearing loss. However, accumulating evidence suggests that psychological distress, emotional reactivity, and attentional control also play significant roles in symptom severity and persistence [2]. Recent models of tinnitus pathophysiology incorporate auditory deafferentation and central nervous system mechanisms, including altered limbic-auditory interactions and maladaptive plasticity [3]. More recently, studies have highlighted the strong interplay between tinnitus and mental health. Jiang et al. [4], for example, demonstrated robust links between tinnitus severity and depression, anxiety, stress, and suicidal ideation, attributing these associations to altered limbic activity and dysregulation of the hypothalamic–pituitary–adrenal (HPA) axis. Such findings provide a neurobiological basis for the heightened emotional distress often reported by individuals with tinnitus.

The COVID-19 pandemic has introduced new complexities into tinnitus management. Increasingly, reports have documented new-onset or worsening tinnitus following SARS-CoV-2 infection and, to a lesser extent, after COVID-19 vaccination [5,6]. Hypothesized mechanisms include neuroinflammatory effects, endothelial dysfunction, and dysregulated immune responses, which may compromise auditory structures or central processing [7]. Pandemic-related psychosocial stress, isolation, and reduced access to hearing healthcare may also exacerbate tinnitus symptoms [8,9]. Indeed, recent work emphasizes that stress may act as a potent trigger or amplifier of tinnitus via psychological and neurobiological pathways rather than through direct alterations in auditory physiology [10].

In addition to central and emotional mechanisms, peripheral auditory factors remain critical to understanding tinnitus. Tinnitus is often associated with cochlear damage and sensorineural hearing loss, reflecting dysfunction of both outer and inner hair cells and altered neural input to the auditory cortex. A recent study by Maihoub, Mavrogeni, Molnár, and Molnár [11] demonstrated that speech recognition thresholds strongly correlate with tinnitus intensity in individuals with primary subjective tinnitus, suggesting that tinnitus severity can directly influence speech comprehension even when standard audiometric thresholds remain within normal limits. Their findings further emphasized that primary tinnitus frequently coexists with sensorineural hearing loss and may also arise from systemic factors, including metabolic, vascular, and inflammatory dysfunctions, which collectively contribute to auditory and perceptual disturbances. These results highlight the importance of assessing speech perception alongside pure-tone thresholds to better understand functional communication challenges in patients with tinnitus.

While anecdotal reports have linked both SARS-CoV-2 infection and vaccination to tinnitus onset, systematic investigations using validated instruments are limited. Tai, Jain, Kim, and Husain [12] provided one of the most comprehensive examinations to date, showing that tinnitus burden is elevated among individuals who report onset or worsening after SARS-CoV-2 infection, vaccination, or pandemic-related stress. These findings suggest that tinnitus in this context is not qualitatively distinct but reflects the broader patterns of emotional distress, functional impairment, and variability commonly observed in tinnitus populations. Prior studies have also underscored the importance of assessing functional hearing outcomes, including speech-in-noise perception and listening effort, which are often impaired in individuals with tinnitus [13,14].

The current study builds on this emerging literature by using standardized questionnaires, including the Tinnitus Functional Index (TFI; [15]), the Speech, Spatial and Qualities of Hearing Scale (SSQ; [16]), and visual analog scales, to examine self-reported tinnitus severity and communication difficulties in individuals reporting tinnitus after COVID-19 vaccination. By situating these results within the broader context of pandemic-related stress and known tinnitus mechanisms, this online multi-domain study aims to characterize the perceptual, functional, and emotional consequences of tinnitus in this population and provide clinical insights into individualized assessment and management approaches.

## 2. Methods

### 2.1. Survey Design

A structured online survey was developed to characterize the experiences of individuals reporting tinnitus after COVID-19 vaccination.

The survey included questions on demographics, vaccination history, tinnitus characteristics, medical and family history, and the impact of pandemic-related factors on auditory symptoms. Additional sections asked about prior audiological evaluations, imaging and laboratory testing, and tinnitus management strategies.

Validated measures were embedded within the survey to assess symptom severity and functional outcomes. These included the TFI [15] and the Tinnitus Reaction Questionnaire (TRQ; [17]), visual analogue numeric rating scales for loudness discomfort and hyperacusis-related symptoms, and the 12-item Speech, Spatial and Qualities of Hearing Scale (SSQ; [16]) to evaluate perceived communication difficulties.

### 2.2. Data Collection

Data was collected in two phases. Phase 1 (August–October 2021) recruited participants through the Facebook group “Tinnitus and Hearing Loss/Impairment after COVID Vaccination,” a public support community for individuals reporting auditory issues post-vaccination. Because this recruitment approach may attract individuals who are already experiencing auditory symptoms, we acknowledge this as a potential source of self-selection bias, as further discussed in the Limitations section. The second phase took place at San José State University between December 2022 and May 2023, using a refined survey instrument that placed greater emphasis on vaccination timelines, medical and noise-exposure history, and perceptual changes in tinnitus. Data from both phases (Phase 1: n = 456; Phase 2: n = 314) were combined for analysis following data cleaning and consistency checks.

Participation in both phases was voluntary, and electronic informed consent was obtained before the start of the survey. Eligibility criteria required that participants (1) confirm receipt of at least one COVID-19 vaccination, and (2) self-report either new-onset tinnitus or a worsening of pre-existing tinnitus temporally associated with vaccination. Exclusion criteria included self-reported neurological disorders (e.g., epilepsy, multiple sclerosis), major psychiatric illness (e.g., schizophrenia, bipolar disorder), and active otologic conditions such as Ménière’s disease or acute otitis media. No control group was included (e.g., individuals with tinnitus unrelated to vaccination or tinnitus after SARS-CoV-2 infection), as the objective of this study was descriptive rather than comparative.

### 2.3. Data Cleaning

A total of 840 survey responses were collected across both phases. Of these, 46 incomplete responses were excluded, along with four duplicate entries and four responses deemed inaccurate or inconsistent. The final analytic sample consisted of 770 complete and valid responses.

### 2.4. Statistical Analysis

Descriptive statistics were used to summarize demographic, vaccination, and tinnitus-related variables. The normality of continuous variables was assessed using the Shapiro–Wilk test, which indicated that the data were not normally distributed. Consequently, nonparametric tests were applied: chi-square tests for categorical variables and Kruskal–Wallis or Mann–Whitney U tests for continuous variables, as appropriate. Spearman correlation analyses were performed to explore associations among tinnitus severity, communication outcomes, and hyperacusis-related symptoms. All analyses were conducted using IBM SPSS Statistics, Version 29.0 (IBM Corp., Armonk, NY, USA) and R version 4.3.

## 3. Results

### 3.1. Participant Characteristics

**Figure 1:**
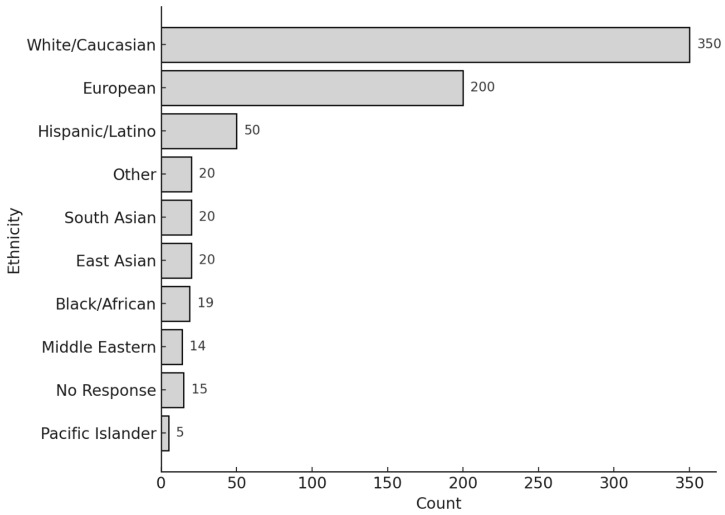
Distribution of self-reported ethnicity among study participants (N = 770). The majority identified as White/Caucasian or European American. Bar heights indicate the number of respondents per category, highlighting the ethnic composition of the study sample.

The average age of respondents was 54.7 years (SD = 14.4). Gender distribution included 450 females, 303 males, and 17 individuals who either selected “Other” or did not specify their gender (Table 1). Respondents identified with the following ethnic backgrounds: White/Caucasian (356), European American (291), Hispanic/Latino (62), Black/African American (10), South Asian (21), East Asian (16), American Indian/Alaskan Native (8), Middle Eastern (8), Pacific Islander (1), Other (28), and No Response (2) (Figure 1). Of the 770 respondents, 48 reported a SARS-CoV-2 infection, with only one hospitalization.

All analyses presented herein pertain to self-reported tinnitus onset or exacerbation that occurred subsequent to COVID-19 vaccination.

### 3.2. Vaccination Profiles

Table 2 outlines the distribution of vaccine types among all survey participants and specifically U.S.-based respondents. Pfizer was the most received vaccine, followed by Moderna.

**Figure 2:**
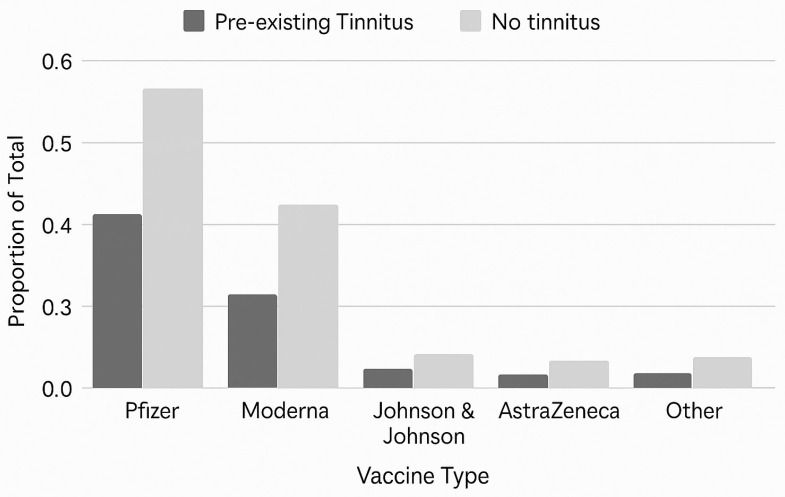
Distribution of COVID-19 vaccine types among individuals with and without pre-existing tinnitus. Each bar shows the number of participants receiving a given vaccine type, segmented by tinnitus status. Data from both recruitment phases (Phase 1: *n* = 456; Phase 2: *n* = 314) were combined for all analyses after ensuring comparable variable structure and completeness. Pfizer and Moderna vaccines were most commonly received across both groups.

Figure 2 displays the proportion of vaccine types received among individuals with and without pre-existing tinnitus. Across both groups, Pfizer and Moderna vaccines were the most administered, and the distribution patterns were broadly similar, with only slight proportional variations between participants with and without pre-existing tinnitus. Participants without tinnitus were somewhat more likely to have received Pfizer (56% vs. 42%), while Moderna showed a similar, though less pronounced pattern (43% vs. 32%). Johnson & Johnson, AstraZeneca, and other vaccines accounted for only a small proportion of the sample. Overall, the distribution of vaccine type appeared broadly similar across groups, with only modest differences.

Table 3 summarizes the vaccine types, number of doses received, and presence of pre-existing tinnitus among respondents. These data help distinguish between new-onset tinnitus and cases in which symptoms may have been exacerbated post-vaccination.

### 3.3. Medical and Audiological Follow-Up

Of the 373 individuals who responded to this section, 224 reported undergoing follow-up medical or hearing assessments (Table 4). These included audiometric evaluations (pure-tone audiometry), ear, nose, and throat (ENT) consultations, magnetic resonance imaging (MRI), and Balance tests (video nystagmography (VNG) and, in some cases, caloric testing), bloodwork, and specialist assessments in neurology and related fields. Audiometric evaluations were the most frequently reported.

#### 3.3.1. Tinnitus Functional Index (TFI)

**Figure 3:**
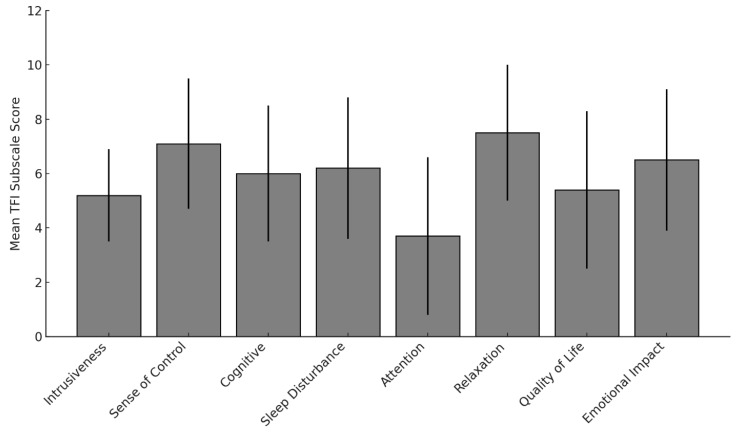
Mean values (± standard deviation) for each of the eight subscales of the Tinnitus Functional Index (TFI). Higher scores indicate greater perceived tinnitus-related burden. The highest average scores were observed in the domains of Relaxation, Sense of Control, and Emotional Impact, while Attention showed the lowest average rating.

Responses to the TFI subscales revealed substantial tinnitus-related burden across multiple life domains (Figure 3). The highest mean scores were observed for Relaxation (M ≈ 7.5), Sense of Control (M ≈ 7.2), and Emotional Impact (M ≈ 6.5). These findings indicate that tinnitus interferes most with emotional regulation, coping, and rest. Attention was rated lowest (M ≈ 3.7), although considerable individual variability was present. Other domains, including Sleep Disturbance, Cognitive effects, and Auditory-related difficulties, also showed moderate-to-high mean scores.

**Figure 4:**
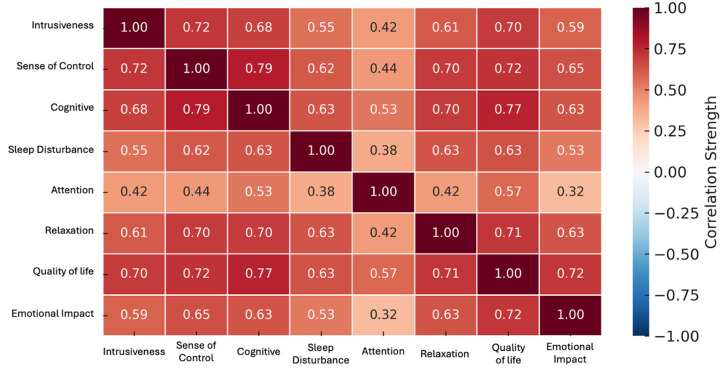
Correlation matrix of Tinnitus Functional Index (TFI) subscales. Each cell represents the Spearman correlation coefficient between two subscales, with stronger correlations indicated by darker shading. Strong positive associations were observed among Cognitive, Sense of Control, Relaxation, and Quality of Life, while Attention showed the weakest correlations with other domains. Note: All correlation coefficients were statistically significant at *p* < 0.05.

Pairwise correlations among the TFI subscales (Figure 4) indicated strong positive associations across most domains. *Sense of Control* was strongly correlated with *Cognitive* (r = 0.79), *Quality of Life* (r = 0.72), and *Relaxation* (r = 0.70). Similarly, *Cognitive* showed strong correlations with *Quality of Life* (r = 0.77) and *Relaxation* (r = 0.70). Moderate associations were observed between *Intrusiveness* and *Quality of Life* (r = 0.70), *Intrusiveness* and *Sense of Control* (r = 0.72), and *Sleep Disturbance* and *Relaxation* (r = 0.63). The weakest correlation was between *Attention* and *Emotional Impact* (r = 0.32), though still statistically significant (*p* < 0.05), suggesting that even less-related domains share variance.

#### 3.3.2. Intrusiveness and Sense of Control

A substantial proportion of respondents reported constant awareness of tinnitus, with 35% perceiving it during 100% of waking hours. Loudness ratings were concentrated at the higher end of the scale, and many participants described minimal perceived control, with 30% indicating tinnitus was “impossible to ignore”.

#### 3.3.3. Cognitive, Sleep, and Emotional Burden

Many respondents endorsed severe interference with concentration and clear thinking, as well as significant sleep disruption. More than one-third reported persistent anxiety, distress, or depressive symptoms linked to their tinnitus.

#### 3.3.4. Auditory and Communication Interference

**Figure 5:**
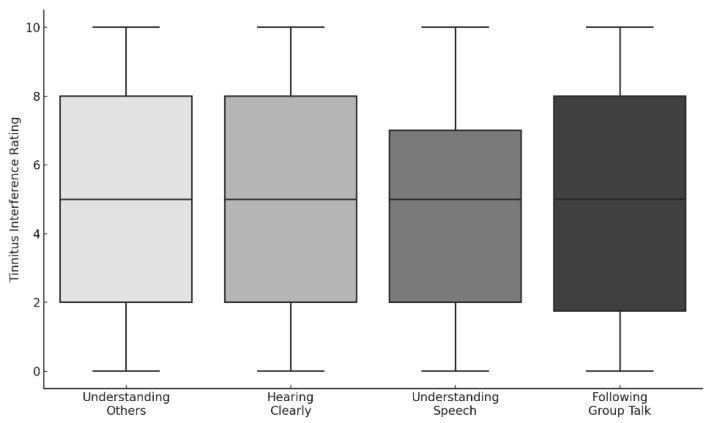
Boxplots depicting self-reported tinnitus interference ratings (scale: 0 = no interference to 10 = extreme interference) across four communication domains: understanding others, hearing clearly, understanding speech, and following group talk. Median interference levels were comparable across all domains.

While many respondents reported preserved hearing clarity, difficulties were more pronounced in speech understanding and following group conversations. Interference ratings across communication domains were moderately elevated (median ≈ 5) and did not differ significantly across conditions (Figure 5).

#### 3.3.5. Hyperacusis and Loudness Sensitivity

**Figure 6:**
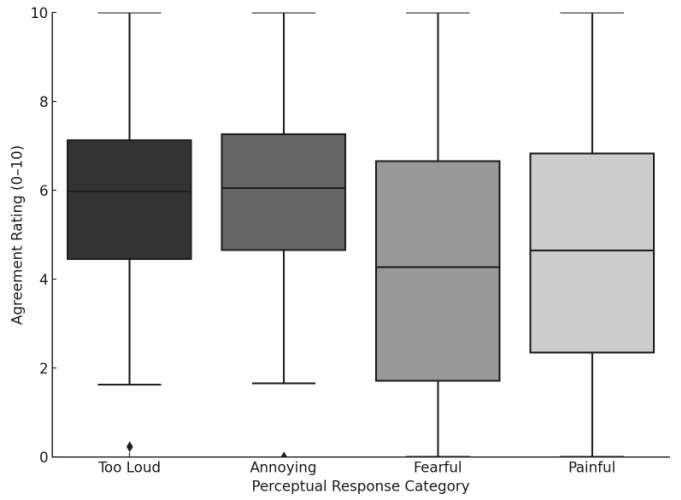
Boxplots of self-reported agreement ratings (0–10) across four perceptual domains related to sound tolerance among individuals reporting tinnitus after COVID-19 vaccination. Participants responded to whether moderately loud sounds were perceived as too loud, annoying, fear-inducing, or painful. Median agreement was highest for “Too Loud” and “Annoying”.

Elevated sound sensitivity was commonly reported. Participants endorsed statements such as “moderately loud sounds are too loud” and “everyday sounds are painful,” consistent with hyperacusis-related symptoms (Figure 6). Agreement ratings were broadly distributed, suggesting variability in sound tolerance, but no statistically significant differences were found across items.

#### 3.3.6. Functional Hearing Outcomes (SSQ)

**Figure 7:**
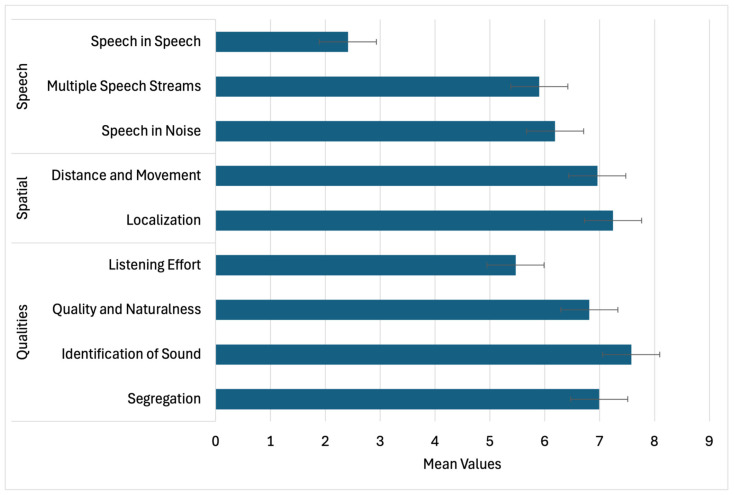
Mean ratings (± standard deviation) for the Speech, Spatial, and Qualities subdomains of the Speech, Spatial, and Qualities of Hearing Scale (SSQ). Participants reported the lowest average performance in the Speech domain, particularly under multi-talker conditions.

SSQ ratings indicated challenges primarily in speech-related conditions (Figure 7). The lowest ratings were observed for “Speech in Speech” (M ≈ 3.5) and other multi-talker or noisy environments (M ≈ 6.0). Spatial hearing (localization, distance, movement) was rated more favorably, with means above 7.0, while the Qualities domain showed strong performance in sound identification and segregation but lower scores for listening effort (M ≈ 5.2). Item-level analyses confirmed significant clustering across domains (Table 5). Strong intercorrelations were observed between Speech, Spatial, and Qualities domains (r = 0.73–0.81; Table 6), underscoring the interconnected nature of functional hearing outcomes.

#### 3.3.7. Loudness Ratings and Lateralization

On a visual analog scale, the most common rating for current tinnitus loudness was 6/10, while 24% reported a maximum loudness of 10/10 during their worst episode. Perceived location was most often the left ear (31.7%), followed by the center of the head (28.4%) and right ear (22.4%). A consistent left-ear predominance was observed for unilateral cases.

## 4. Discussion

This study investigated the impact of SARS-CoV-2 infection and vaccination on tinnitus, focusing on self-reported changes in perceptual features (e.g., loudness, pitch, location), emotional and cognitive functioning, and speech-in-noise perception. Using validated instruments, including the TFI, SSQ, and visual analog rating scales, this study provides a comprehensive profile of tinnitus burden during the pandemic. Key findings include elevated distress in TFI subdomains such as Relaxation and Emotional Impact, consistent left-ear lateralization, reports of worsened tinnitus following vaccination, and increased listening effort in noisy environments. Additionally, high correlations between SSQ domains suggest interconnected auditory processing difficulties.

These findings align with an expanding body of post-pandemic research emphasizing perceptual variability, emotional comorbidity, and auditory sensitivity in individuals with tinnitus. Yellamsetty [18] documented increases in tinnitus loudness and hyperacusis following COVID-19 vaccination, while Yellamsetty and Gonzalez [9] highlighted pandemic-related psychosocial stressors as exacerbating factors. Wang et al. [19] identified pre-vaccination metabolic vulnerabilities that may predispose individuals to auditory disturbances. A comprehensive survey by Yellamsetty, Egbe-Etu, and Bao [20] confirmed symptom heterogeneity across a large cohort. These patterns echo findings from Beukes et al. [5], who reported worsening tinnitus severity, increased emotional distress, and disrupted healthcare access during the pandemic, and from Figueiredo et al. [6], who reported variable tinnitus outcomes following vaccination. Importantly, Tai et al. [12] conducted a systematic investigation of tinnitus in relation to SARS-CoV-2 infection, vaccination, and pandemic stress, concluding that tinnitus burden is elevated across these groups and not limited to a single etiology. The present findings extend this literature by characterizing a large sample of individuals who attribute tinnitus onset or exacerbation specifically to vaccination, while recognizing that the symptom burden is consistent with that observed in tinnitus more broadly.

### 4.1. Tinnitus-Related Impairments Across Functional Domains

In the context of normative data, the present findings indicate a clinically significant level of tinnitus-related distress among individuals who report new or worsening symptoms after COVID-19 vaccination. Participants’ mean *Sense of Control* score on the TFI (M = 7.2) was markedly higher than values typically observed in non-tinnitus control groups (M ≈ 1.5–2.0; [15]), signifying a substantial loss of perceived control and difficulty coping with tinnitus. Likewise, mean *Speech* and *Spatial* subscale scores on the SSQ (M ≈ 6.0–7.0) were below normative benchmarks for adults with normal hearing (M ≈ 8.5–9.0; [16]), suggesting notable challenges in everyday communication, particularly in complex listening environments. Together, these comparisons confirm that the symptom burden in this cohort is clinically meaningful and functionally limiting, aligning with the profile of individuals who experience persistent or bothersome tinnitus.

Across domains, the TFI results underscore the multidimensional nature of tinnitus-related distress, with the greatest impact in Relaxation, Emotional Distress, and Sense of Control. A substantial proportion of participants reported constant awareness, high loudness ratings, and persistent annoyance, consistent with previous work showing the close association between tinnitus severity and psychological comorbidities [2,21]. Reduced sense of control and difficulty ignoring tinnitus reinforce the interplay between perceptual and emotional domains, while sleep disturbance and cognitive interference, well-established correlates of tinnitus [15], were also frequently endorsed.

While some participants reported little disruption in hearing clarity, qualitative and quantitative data highlighted difficulties with group communication and speech understanding, consistent with reports of listening fatigue in complex environments. These findings parallel more recent pandemic-era research documenting heightened distress, reduced access to care, and increased auditory sensitivity as contributors to worsened tinnitus outcomes [5,22]. Collectively, the results support prior literature while providing descriptive evidence from individuals who report post-vaccination tinnitus.

Moreover, our results contribute to the growing body of literature identifying strong associations between tinnitus and mental health challenges, particularly during global crises. Jiang et al. [4] recently demonstrated robust links between tinnitus severity and depression, anxiety, stress, and suicidal ideation. Their findings of altered limbic activity and hypothalamic–pituitary–adrenal (HPA) axis dysregulation provide a plausible neurobiological basis for the sustained increases in tinnitus distress observed in our cohort. Recent work by Maihoub et al. [11] further emphasizes that pandemic-related stress can act as a potent trigger or amplifier of tinnitus through psychological and neurobiological pathways, rather than through direct alterations of peripheral auditory physiology. These insights resonate with our findings of heightened emotional impact and sense-of-control difficulties, suggesting that stress-mediated mechanisms may play a significant role in shaping tinnitus burden during and after the pandemic. Recent population-level analyses further demonstrate that tinnitus distress fluctuated across the course of the COVID-19 pandemic. Yellamsetty and Shin [23] found that symptom severity increased during periods of heightened community stress and uncertainty, suggesting that broader psychosocial conditions can meaningfully shape tinnitus trajectories. These findings complement the present results, reinforcing the interpretation that emotional burden, reduced sense of control, and stress-mediated pathways are central contributors to tinnitus severity during pandemic and post-vaccination contexts.

Compared to prior investigations that have primarily examined tinnitus linked to SARS-CoV-2 infection (e.g., Beukes et al. [5]; Viola et al. [7]) or vaccination in isolation (e.g., Yellamsetty et al., [18]), the present study contributes by using validated instruments (TRQ, TFI, SSQ) to capture tinnitus severity and functional impact across multiple pandemic-related contexts. This design provides a broader perspective on both psychosocial and vaccine-related influences, enabling a more comprehensive characterization of tinnitus trajectories. This divergence underscores the need for future research to disentangle stress-mediated mechanisms from peripheral auditory effects when examining tinnitus during and after the pandemic.

### 4.2. Speech Perception and Listening Effort in Complex Environments

SSQ results indicated that spatial hearing abilities (e.g., localization, distance estimation) were largely preserved, while speech-in-noise and multi-talker conditions were rated as particularly challenging. Elevated listening effort was consistently endorsed, reflecting the increased cognitive load required to maintain communication. These findings mirror established evidence that tinnitus is associated with greater attentional demand and reduced efficiency in auditory scene analysis [13,14].

Hyperacusis-related symptoms also emerged, with participants reporting excessive loudness, annoyance, and in some cases fear or pain in response to everyday sounds. These results are consistent with previous findings of increased sound sensitivity among individuals with tinnitus [20,24,25]. The strong correlations among SSQ domains reinforce that these functional communication difficulties are interconnected and likely reflect shared perceptual and cognitive mechanisms, a conclusion also supported by Beukes et al. [5].

### 4.3. Perceived Loudness and Lateralization

Self-reported tinnitus loudness revealed a bimodal distribution, with moderate loudness commonly reported during daily life and extreme loudness endorsed by nearly one-quarter of respondents at its worst. This finding may suggest partial adaptation over time, though residual burden remained high. Lateralization patterns showed a consistent left-ear predominance, consistent with previous clinical and neuroimaging studies implicating hemispheric auditory asymmetries. The high proportion of reports of centralized or diffuse tinnitus highlights the need for careful characterization of perceptual localization in both clinical and research contexts.

### 4.4. Mechanisms Underlying COVID-19 Vaccination-Related Tinnitus

The biological mechanisms underlying tinnitus following COVID-19 vaccination remain uncertain, but accumulating evidence supports a multifactorial framework involving immune, vascular, neuroinflammatory, and psychosocial processes. In the present study, a subset of respondents reported perceptible changes in tinnitus characteristics—including increased loudness, pitch, and emotional reactivity—within days of vaccination, often accompanied by hyperacusis and communication difficulties. Comparable findings have been documented in observational cohorts, with an estimated 5–15% of individuals reporting new or worsened tinnitus following vaccination [5,6,22]. While immune activation has been proposed as a primary mechanism, our results do not support an overactive systemic immune response as the main cause. The temporal mismatch between the onset of tinnitus (often within two days) and the slower systemic immune response to vaccination, as well as the higher incidence after the first rather than subsequent doses, suggests that other mechanisms may be involved.

One possible pathway involves spike protein–mediated neurovascular effects. Experimental studies show that spike proteins can disrupt the blood–brain barrier [26,27,28], activate microglia and neuroinflammatory cascades [4,29], and induce neuronal damage or misfolded protein aggregation [29]. These processes may transiently alter neural excitability or auditory gain control, potentially contributing to tinnitus perception. Additionally, rapid systemic inflammatory responses secondary to vaccine reactogenicity and endothelial dysfunction or vascular inflammation, including in cerebral and cochlear microcirculation [30,31], could disrupt auditory or limbic network regulation.

Other pathways may act at the peripheral level of the auditory system. The human inner ear expresses angiotensin-converting enzyme-2 (ACE2) receptors—the binding site for the SARS-CoV-2 spike protein [32], suggesting that spike protein interactions could transiently affect cochlear or vestibular function. However, because most respondents did not report measurable hearing loss, inner ear dysfunction alone is unlikely to explain the observed tinnitus. Theoretical concerns about mRNA translation errors or off-target protein products [33] remain speculative and are unlikely to account for tinnitus cases associated with non-mRNA vaccines.

Beyond direct biological mechanisms, psychological and neuroendocrine factors may amplify tinnitus burden. Pandemic-related stress, uncertainty, and vaccine-associated anxiety can activate the hypothalamic–pituitary–adrenal (HPA) axis and increase limbic system reactivity, thereby heightening tinnitus perception and distress [4,11]. Similarly, pre-existing anxiety, metabolic vulnerability, or auditory sensitivity may predispose certain individuals to experience tinnitus onset or exacerbation after vaccination or infection.

Taken together, current evidence supports a multifactorial etiology in which immune and neuroinflammatory signaling, vascular or endothelial dysfunction, and stress-mediated pathways interact to influence tinnitus expression and persistence in the context of COVID-19 vaccination, infection, and pandemic-related stress.

### 4.5. Clinical and Research Implications

This study reinforces the need for a multidimensional assessment of tinnitus that incorporates both symptom severity and functional communication outcomes. Tools such as the TFI and SSQ are valuable for capturing the emotional, perceptual, and cognitive consequences of tinnitus. Clinically, audiologists should integrate counseling and cognitive support, as psychological distress and attentional mechanisms strongly influence patient experiences. From a research perspective, the findings highlight the importance of including control groups and longitudinal follow-up in future studies to better delineate whether tinnitus associated with vaccination differs from tinnitus triggered by infection or pandemic stress, as emphasized by Tai et al. [12].

### 4.6. Limitations and Future Directions

Several limitations should be acknowledged. The study relied on self-reported data, with no objective audiometric verification. Recruitment through online surveys and support groups introduces potential selection bias. Most importantly, the study did not include a control group (e.g., individuals with tinnitus unrelated to vaccination or those developing tinnitus after infection), limiting the ability to determine whether the findings are specific to post-vaccination tinnitus or reflect broader tinnitus populations.

Because the first phase of data collection was recruited from a tinnitus support group focused on auditory symptoms after vaccination, there is a possibility of selection bias toward individuals with more severe or persistent tinnitus. Future studies should utilize broader population-based recruitment strategies to validate these findings. Nonetheless, the large sample size and use of validated measures strengthen the descriptive value of the results. Future work should incorporate longitudinal designs, biological and neuroimaging markers, and comparison groups to clarify mechanisms. Integrating insights from Tai et al. [12] and others, future research should aim to disentangle the relative contributions of infection, vaccination, and psychosocial stress to tinnitus burden.

## 5. Conclusions

This study contributes to the growing body of evidence documenting the multidimensional burden of tinnitus during the COVID-19 pandemic. Individuals reporting tinnitus after vaccination described significant emotional distress, cognitive interference, sleep disruption, and communication difficulties, patterns consistent with those observed in individuals with bothersome tinnitus more broadly. While causality cannot be established, the findings highlight the need for comprehensive clinical evaluation and patient-centered management approaches that integrate both audiological and psychological assessment. Emerging evidence and the present data together support a multifactorial framework, suggesting that immune-mediated inflammation, neurovascular and neuroinflammatory processes, and stress-related neuroendocrine dysregulation may collectively contribute to the onset or exacerbation of tinnitus following COVID-19 vaccination. Future controlled, longitudinal studies incorporating objective auditory and biological measures are needed to better elucidate these mechanisms and identify individuals at increased risk.

## Figures and Tables

**Table 1:** Demographics of survey respondents.

Characteristics	Category	n (%)
Age (years)	Mean = 54.7 ± 14.4 (SD); Range = 18–85	—
Gender	Female	450 (58.4%)
	Male	303 (39.4%)
	Other/Unspecified	17 (2.2%)
COVID-19 Infection History	Reported SARS-CoV-2 infection	48 (6.2%)
	Hospitalized due to infection	1 (0.1%)

**Table 2:** Frequency and distribution of tinnitus cases across vaccine manufacturers. Includes total responses and U.S.-based cases. Pfizer and Moderna were the most common vaccines received among participants.

Vaccine Manufacturer	All Survey Cases (n = 770)	U.S. Survey Cases (n = 618)
Pfizer	427 (55.5%)	328 (53.1%)
Moderna	256 (33.2%)	239 (38.7%)
Johnson & Johnson	55 (7.1%)	48 (7.8%)
AstraZeneca	28 (3.6%)	2 (0.3%)
Other	4 (0.5%)	1 (0.2%)

**Table 3:** Dose series by vaccine manufacturer and pre-existing tinnitus. Includes the number of participants who received 1, 2, 3, or 4+ doses for each manufacturer, with a breakdown by pre-existing tinnitus status.

Vaccine Manufacturer	1 Dose	2 Doses	3 Doses	4+ Doses	Total Cases (n = 766)	Pre-Existing Tinnitus (n, %)
Pfizer	16 (3.7%)	187 (43.8%)	177 (41.5%)	47 (11.0%)	427	113 (26.5%)
Moderna	7 (2.7%)	94 (36.7%)	122 (47.7%)	33 (12.9%)	256	67 (26.2%)
Johnson & Johnson	31 (56.4%)	10 (18.2%)	10 (18.2%)	4 (7.3%)	55	17 (30.9%)
AstraZeneca	18 (64.3%)	8 (28.6%)	2 (7.1%)	0 (0%)	28	4 (14.3%)

**Table 4:** Medical evaluations following tinnitus onset. Includes frequencies of audiological and neurological assessments among 373 participants, segmented by gender where available.

Type of Evaluation	Total (n = 373)	Male (n = 162)	Female (n = 199)	Other/Unspecified (n = 12)
Audiometry (PTA)	161 (43.2%)	73 (45.1%)	85 (42.7%)	3 (25.0%)
ENT Consultation	105 (28.2%)	52 (32.1%)	50 (25.1%)	3 (25.0%)
MRI (Brain)	89 (23.8%)	40 (24.7%)	46 (23.1%)	3 (25.0%)
Blood Tests	54 (14.5%)	26 (16.0%)	27 (13.6%)	1 (8.3%)
Vestibular VNG/caloric Tests	27 (7.2%)	12 (7.4%)	14 (7.0%)	1 (8.3%)
Neurological test	26 (7.0%)	13 (8.0%)	12 (6.0%)	1 (8.3%)
Other (e.g., TMJ, Cardiology)	18 (4.8%)	7 (4.3%)	10 (5.0%)	1 (8.3%)

**Table 5:** Chi-square test results for Speech, Spatial, and Qualities of Hearing Scale (SSQ) item responses. All items demonstrated significant deviation from uniform distributions, indicating patterned responses across domains.

SSQ Domain	Item Description	χ^2^ (df)	*p*-Value
Speech	Speech in Noise (TV on)	125.61	<0.001
	Multiple Streams (TV & person)	57.89	<0.001
	Speech in Speech (Crowd)	67.16	<0.001
	Group Speech (Restaurant)	55.33	<0.001
	Switching Speakers	88.26	<0.001
Spatial	Localization (Dog barking)	248.56	<0.001
	Distance Judgement	168.11	<0.001
	Directionality (Coming vs. Going)	188.84	<0.001
Qualities	Segregation (Jumbled Sounds)	272.88	<0.001
	Identifying Instruments	311.27	<0.001
	Sound Naturalness (Clarity)	165.83	<0.001
	Listening Effort	46.94	<0.001

**Table 6:** Spearman correlation matrix across the three SSQ domains (Speech, Spatial, Qualities). Strong correlations were observed among all domains, particularly between Speech and Qualities (r = 0.81).

	Speech	Spatial	Qualities
Speech	1.00	0.73 ***	0.81 ***
Spatial	—	1.00	0.77 ***
Qualities	—	—	1.00

Note: *** signifies *p* < 0.001.

## Data Availability

The original contributions presented in the study are included in the article, further inquiries can be directed to the corresponding author.
